# The ****Role of Properdin in Killing of Non-Pathogenic *Leptospira biflexa*

**DOI:** 10.3389/fimmu.2020.572562

**Published:** 2020-11-10

**Authors:** Adriana Patricia Granados Martinez, Patrícia Antonia Estima Abreu, Silvio de Arruda Vasconcellos, Paulo Lee Ho, Viviana P. Ferreira, Gurpanna Saggu, Angela Silva Barbosa, Lourdes Isaac

**Affiliations:** ^1^ Department of Immunology, Institute of Biomedical Sciences, University of São Paulo, São Paulo, Brazil; ^2^ Laboratory of Bacteriology, Butantan Institute, São Paulo, Brazil; ^3^ Laboratory of Bacterial Zoonoses, Faculty of Veterinary Medicine and Animal Science, University of São Paulo, São Paulo, Brazil; ^4^ Department of Medical Microbiology and Immunology, College of Medicine and Life Sciences, University of Toledo, Toledo, OH, United States

**Keywords:** properdin, *Leptospira*, complement system, alternative pathway, bacteria killing ability

## Abstract

Properdin (P) is a positive regulatory protein that stabilizes the C3 convertase and C5 convertase of the complement alternative pathway (AP). Several studies have suggested that properdin can bind directly to the surface of certain pathogens regardless of the presence of C3bBb. Saprophytic *Leptospira* are susceptible to complement-mediated killing, but the interaction of properdin with *Leptospira* spp. has not been evaluated so far. In this work, we demonstrate that properdin present in normal human serum, purified properdin, as well as properdin oligomers P2, P3, and P4, interact with *Leptospira*. Properdin can bind directly to the bacterial surface even in the absence of C3b. In line with our previous findings, AP activation was shown to be important for killing non**-**pathogenic *L. biflexa*, and properdin plays a key role in this process since this microorganism survives in P-depleted human serum and the addition of purified properdin to P-depleted human serum decreases the number of viable leptospires. A panel of pathogenic *L.* *interrogans* recombinant proteins was used to identify putative properdin targets. Lsa30, an outer membrane protein from *L. interrogans*, binds to unfractionated properdin and to a lesser extent to P2-P4 properdin oligomers. In conclusion, properdin plays an important role in limiting bacterial proliferation of non-pathogenic *Leptospira* species. Once bound to the leptospiral surface, this positive complement regulatory protein of the AP contributes to the formation of the C3 convertase on the leptospire surface even in the absence of prior addition of C3b.

## Introduction

Spirochetes of the genus *Leptospira* may cause leptospirosis, a zoonosis of worldwide distribution. The *Leptospira* genus includes pathogenic and saprophytic species, which are classified into more than 300 serovars (1). Pathogenic leptospires have evolved virulence strategies to successfully colonize a variety of hosts, but the mechanisms of pathogenesis in leptospirosis are still poorly defined. While progress has been made in this field, gaps remain in our understanding of how *Leptospira* causes disease.

One of the factors that contribute to a successful infection by spirochetes is their ability to escape the natural defense mechanisms of the human host by circumventing complement-mediated killing. Pathogenic *Leptospira* spp. control complement activation on their surfaces or in the surrounding microenvironment by *i)* hijacking soluble regulatory proteins from the host; *ii)* cleaving complement molecules of the alternative (AP), the lectin and the classical pathways through the secretion of proteases such as thermolysins; or, *iii)* the acquisition of host proteases such as plasminogen [reviewed in (2)]. In contrast, saprophyte *Leptospira* spp. are devoid of complement evasion strategies and so are highly susceptible to the bactericidal activity of human serum (2–4).

A number of surface-exposed proteins have been shown to contribute to complement immune evasion by pathogenic *Leptospira*. By binding soluble complement regulatory proteins that avoid complement activation (negative regulators) such as Factor H, C4b binding protein, and vitronectin, these bacterial proteins potentially down-regulate all three complement pathways as well as the terminal step of this cascade (3–5). While the interaction of these complement regulatory proteins with different *Leptospira* species has been well explored over the last years, binding of properdin, the only positive regulator of the complement cascade, to the surface of saprophyte and pathogenic *Leptospira* has never been evaluated.

Human properdin is a 53 kDa plasma glycoprotein composed of seven tandem repeats called thrombospondin type 1 modules (6, 7). In the circulation, identical subunits display a head to tail arrangement to form dimers, trimers and tetramers in a fixed ratio, the trimers being the most abundant form (8, 9). Properdin is synthesized primarily by neutrophils, dendritic cells, monocytes, macrophages and T cells [reviewed in (10)], and its serum concentration is approximately 5–25 µg/ml (10, 11). A number of important functions have been ascribed to properdin [reviewed in (12). The first studies assessing the role of this complement regulator date back to the 1950s when Pillemer and colleagues demonstrated that properdin could bind to a variety of targets in the absence of specific antibodies and activate the complement system (13).

In the late 1970s, properdin regained attention when a model for the assembly of C3 convertase of the AP emerged. According to this model, today known as the “standard model”, C3b produced by C3 activation in the fluid-phase binds covalently to target surfaces. Subsequently, Factor B associates with C3b, and is then cleaved by Factor D. Factor B amino-terminal fragment (Ba) is released and the C3bBb complex is recognized and stabilized by properdin (14, 15). As a consequence, the C3 convertase half-life is extended by 10-fold or more (16). More recently, a series of studies indicated that physiological forms of properdin can initiate complement activation *in vitro* by directly attaching non-covalently to certain target surfaces, including *Chlamydia pneumoniae*, late apoptotic and necrotic cells, and activated platelets [reviewed in (10)]. Consistent with the “properdin-directed model”, properdin binds to a target surface and recruits C3b. The C3bBb complex is then formed upon association of Factor B with C3b, as previously mentioned.

Given the importance of the AP in eliminating non**-**pathogenic *Leptospira*, we addressed the following questions in the present work: *i)* do pathogenic and non**-**pathogenic leptospiral strains interact differentially with properdin? and *ii)* does properdin bind directly to the bacterial surface or is previous binding of C3b required?

## Material and Methods

### *Leptospira* Strains

Saprophytic *L. biflexa* serovar Patoc strain Patoc I (non-pathogenic), *L. interrogans* serovar Pomona strain Pomona, (pathogenic, culture-attenuated), and *L. interrogans* serovar Kennewicki strain Fromm (pathogenic, virulent), were provided by the Laboratory of Bacterial Zoonoses at the Faculty of Veterinary Medicine and Animal Science, University of São Paulo. *Leptospires* were cultured at 29°C for 7 days under aerobic conditions in EMJH (Difco-USA) liquid medium supplemented with 10% *Leptospira* enrichment EMJH (BD-USA) or with 10% pre-enriched inactivated rabbit serum, L-asparagine (0.015%), sodium pyruvate (0.001%), calcium chloride (0.001%), magnesium chloride (0.001%), and peptone (0.03%). Attenuation of the strain Pomona was achieved by successive passages in culture medium and virulence of the strain Fromm was maintained by successive passages in hamsters.

### *Leptospira* Survival in the Serum

*L. biflexa* serovar Patoc strain Patoc I (2–3 x 10^8^ bacteria/ml) cultured in EMJH medium supplemented with bovine serum albumin (BSA) was incubated at 37°C for 2 h with 40% normal human serum (NHS), heat-inactivated NHS (HI-NHS) obtained after incubation of NHS at 56°C for 30 min; properdin-depleted (P-DS) serum (Complement Technology, Inc.) or P-DS supplemented with 5 μg, 10 μg, or 25 μg of commercial purified human properdin (Complement Technology. Inc.) to a final volume of 100 μl. After incubation, *Leptospira* survival was estimated by counting the number of viable bacteria in Petroff-Hausser’s chamber using dark field microscopy, according to (4). The experiments were performed in triplicate. The survival in the presence of each serum was compared to that observed in HI-NHS (100%).

### Interaction of *Leptospira* With Properdin

Cultures of *L. interrogans* serovar Kennewicki strain Fromm, *L. biflexa* serovar Patoc strain Patoc I, and *L. interrogans* serovar Pomona strain Pomona were washed twice with VBS^++^ (1.46 mM sodium barbiturate; 2.5 mM 5,5’diethyl barbituric acid; 144 mM NaCl, pH 7.4 containing 0.83 mM MgCl_2_ and 0.25 mM CaCl_2_). Each suspension containing 5 x 10^8^ *Leptospira*/ml) was incubated with 0, 10, 25, and 50% NHS containing 10 mM EDTA (NHS-EDTA) or equivalent amounts of purified properdin (0, 2.5, 6.2, and 12.5 µg). *Leptospira* samples were also incubated with 40% C3-depleted human serum (C3-DS) (Complement Technology, Inc.) or with NHS-EDTA for 2 h at 37°C, with stirring, in a final volume of 500 μl. In parallel, *Leptospira* suspensions were incubated with 5 μg of human properdin (Complement Technology, Inc.) or 10 μg of human C3b (Complement Technology, Inc.) for 1 h at 37°C with stirring. After three washes with VBS^++^, 10 μg of C3b was added to the bacterial cultures that had been pre-incubated with properdin, and 5 μg of properdin was added to those that had been pre-incubated with C3b. The bacterial suspensions were then washed five times with VBS^++^ and centrifuged at 3,000 x *g* for 10 min at 4°C. The precipitates were resuspended in 0.1 M NaHCO_3_ pH 9 and 100 μl of each sample were immobilized in duplicate on ELISA plate wells for 16 h at 4°C. Next, the wells were washed twice with PBS containing Tween 0.05% (PBS-T) and blocked with 3% BSA in PBS for 2 h at 37°C. The wells were washed three times with PBS-T. Properdin or C3b bound to the bacterial surface were detected with goat anti-properdin (1:2,000) or goat anti-C3 (1:5,000) (both antibodies were purchased from Complement Technologies, Inc.) diluted in PBS. After incubation for 1 h at 37°C and three washes with PBS-T, peroxidase-conjugated anti-goat IgG, diluted 1: 10,000 in PBS, was added for 1 h at 37°C. After three washes the reactions were developed by adding the substrate *o*-Phenylenediamine dihydrochloride (OPD; 0.04%) diluted in citrate-phosphate buffer (pH 5.0) and 0.01% H_2_O_2_. The plates were protected from light for 5 to 10 min and the reaction was quenched by the addition of 50 μl of 4 N H_2_SO_4_. The absorbance was read at 492 nm.

### Interaction of Properdin Oligomers With *Leptospira* Strains and With Leptospiral Outer Membrane Proteins

We assessed binding of pure properdin oligomers, namely high molecular weight aggregates not present in serum (Pn), properdin dimer (P2), trimer (P3), and tetramer (P4) to pathogenic and non-pathogenic leptospires as well as to leptospiral membrane proteins. *L. biflexa* serovar Patoc strain Patoc I (non-pathogenic), *L. interrogans* serovar Pomona strain Pomona (culture attenuated), and *L. interrogans* serovar Kennewicki strain Fromm (virulent) suspensions containing 1 x 10^8^ leptospires were incubated with 5 µg of each oligomer or with unfractionated commercial properdin (P), and the presence of properdin (total volume of 1 ml) was evaluated by ELISA as described above. In addition, with the aim of identifying putative ligands for properdin on the leptospiral membrane, a panel of recombinant proteins from *L. interrogans* serovar Copenhageni 10A [produced essentially as described in (17) was used (**Table 1**). One microgram of each protein was immobilized on ELISA plate wells and incubated with 0.5 µg of properdin. Binding was assessed as described above. Interaction of Lsa30 with properdin oligomers was also evaluated as already described. The data correspond to three independent experiments, using two separate preparations of P2, P3, P4, or Pn. Each experiment was performed in triplicate.

**Table 1:** Recombinant Proteins from *L. Interrogans* Serovar Copenhageni 10A.

Gene	Protein	GenBank	Recombinant protein molecular mass (kDa)
*LIC10325*	HlyX	AAS68952.1	43
*LIC11947*	LcpA	AAS70529.1	20
*LIC12875*	EF-Tu	AAS71428.1	43
*LIC10301*	–	AAS68928.1	13
*LIC10009*	Lp25	AAS68646.1	25
*LIC10507*	–	AAS69128.1	22
*LIC10704*	–	AAS69325.1	23
*LIC11352*	LipL32	AAS69953.1	32
*LIC10465*	LigA-C	AAS69086.1	63
*LIC10464*	LigB-C	AAS69085.1	56
*LIC10464*	LigB-N	AAS69085.1	64
*LIC13305*	–	AAS71847.1	31
*LIC11087*	Lsa30	AAS69694.1	30
*LIC11030*	–	AAS69637.1	35

### Detection of C3 Convertase on the *Leptospira* Surface

An ELISA-based assay with some modifications was employed. All incubations were performed in microtubes including the development of the reactions with OPD. *L. biflexa* serovar Patoc strain Patoc I and *L. interrogans* serovar Kennewicki strain Fromm cultures were centrifuged at 4,500 x *g* for 20 min at 21°C, then washed twice with PBS. Subsequently, the number of bacteria was estimated using dark field microscopy. 2 x 10^8^ bacteria/ml were incubated with 5 μg/ml of commercial properdin for 1 h at 37°C with stirring. The samples were washed three times with PBS and the pellets were incubated with 10% P-DS, NHS, HI-NHS diluted in AP-CFTD (VBS buffer containing 7 mM MgCl_2_, 10 mM EGTA, pH 7.2) buffer at 37°C for 30 min with stirring. After five washes, polyclonal goat anti-Factor B at a 1: 2,000 dilution (Complement Technology, Inc.) in PBS-T containing 1% BSA was added and incubation proceeded for 1 h at 37°C. After three washes with PBS, anti-goat IgG (KPL) diluted 1: 5,000 in PBS-T containing 1% BSA was added to the bacteria and incubated for 1 h at 37°C. Subsequently, three washes were performed, and the reaction was developed with *o*-phenylenediamine dihydrochloride (OPD) substrate (0.04%) diluted in citrate-phosphate buffer (pH 5.0) and 0.01% H_2_O_2_. After 5 min the reaction was stopped with 50 µl of 4N H_2_SO_4_, the samples were transferred to a 96-well plate and the absorbance read at 492 nm.

### Statistical Analysis

Data was analyzed using ANOVA one-way test, using Statgraphics Centurion XVI software. Except when indicated, the variance homogeneity was assessed using Cochran test and when necessary, data logarithmic transformation was used.

## Results

### Properdin Contributes to *Leptospira biflexa* Killing in the Serum

**Figure 1:**
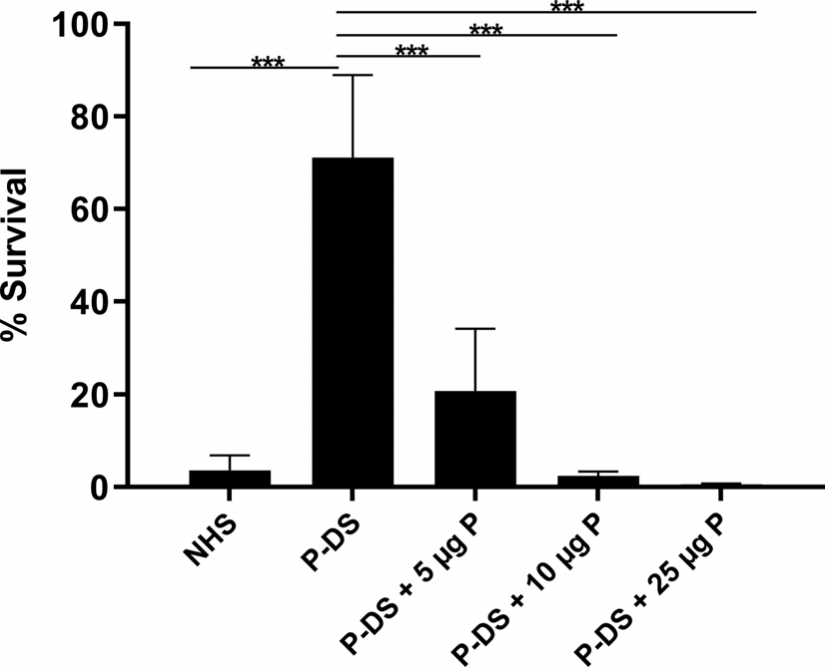
Survival of *L.* *biflexa* serovar Patoc in the presence of properdin. Leptospires were incubated with normal human serum (NHS), properdin-depleted serum (P-DS) and P-DS reconstituted with 5, 10, or 25 µg of purified properdin (P) for 2 h at 37°C. The number of viable leptospires present after incubation with heat inactivated NHS was considered 100%. Data are expressed as the mean ( ± SD) of three independent experiments each one performed in triplicate. Statistically significant differences (ANOVA one-way test) are indicated. ***p ≤ 0.001; confidence interval of 95%.

Considering that the AP is important for the killing of the non**-**pathogenic *L. biflexa* serovar Patoc strain Patoc I (3, 4, 18, 19), we evaluated survival of this strain in P-DS. Under these conditions, 70% of the bacteria survived. When purified properdin (5–25 µg/ml) was added to P-DS, bacterial survival decreased significantly as properdin concentration increased because of stabilization of the C3 convertase (C3bBb) (**Figure 1**). These results further confirm that *L. biflexa* activates the AP, and bacterial elimination depends on the presence of properdin.

### Binding of Properdin to *Leptospira* spp.

**Figure 2:**
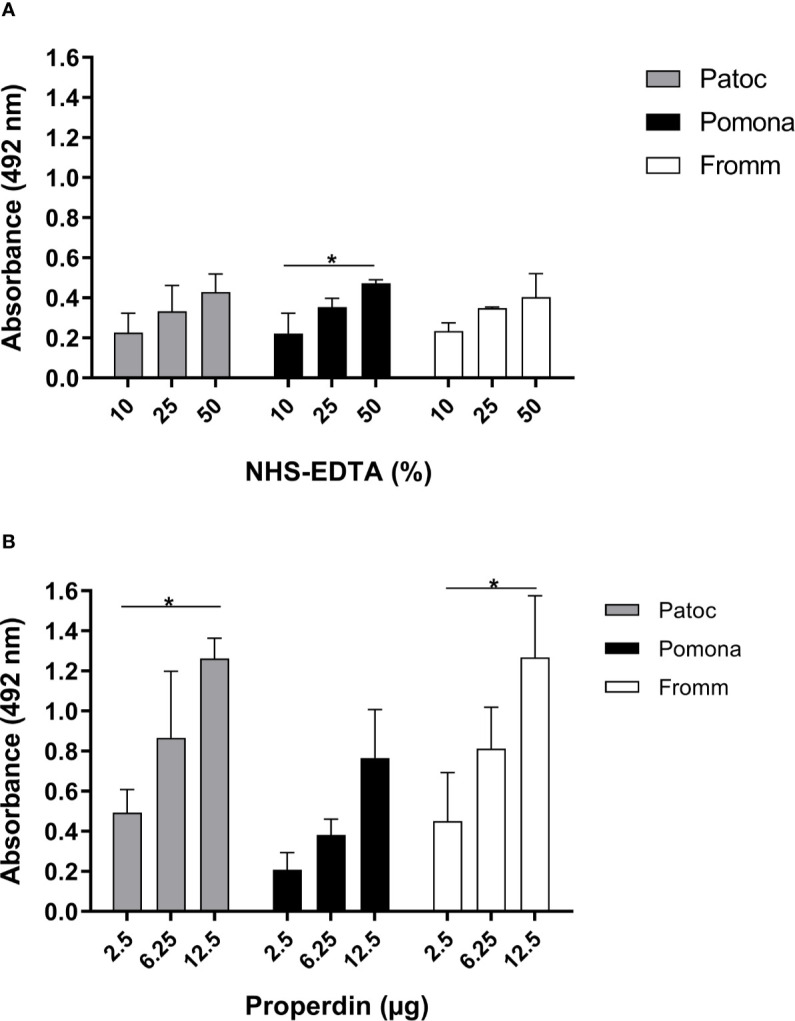
Binding of properdin to *Leptospira.* *L. biflexa* serovar Patoc strain Patoc I (non-pathogenic), *L. interrogans* serovar Pomona strain Pomona (pathogenic, attenuated), and *L. interrogans* serovar Kennewicki strain Fromm (pathogenic, virulent) were incubated with **(A)** 10, 25, and 50% NHS-EDTA or **(B)** 2.5, 6.25, and 12.5 µg of commercial purified properdin which are equivalent to properdin concentrations found in 10, 25, and 50% NHS used above. The binding was evaluated by ELISA using polyclonal anti-human properdin. Baseline values obtained with PBS were subtracted in **(A, B)**, respectively for each type of leptospire. Data are expressed as the mean ( ± SD) of three independent experiments each one performed in triplicate. Statistically significant differences (ANOVA one-way test) are indicated. *p ≤ 0.05; confidence interval of 95%. Variance homogeneity was analyzed using Bartlett.

Next, we investigated if three different species of *Leptospira* including virulent (*L. interrogans* serovar Kennewicki strain Fromm), culture-attenuated (*L. interrogans* serovar Pomona strain Pomona) and non-pathogenic (*L. biflexa* serovar Patoc strain Patoc I) strains, would bind to properdin. To assess this interaction, bacterial cultures were washed and incubated with purified properdin or NHS-EDTA as a source of properdin, and binding was measured by ELISA. Our data shows that properdin, either purified or present in NHS-EDTA, interacts dose-dependently with all *Leptospira* strains tested, regardless of their virulence status (**Figures 2A–B**). Purified properdin binding to leptospires was more pronounced compared to serum properdin binding suggesting a certain degree of competition between properdin and other serum molecules. Another possibility to explain this result would be the presence of inhibitors that may interfere with the properdin binding capacity on the surface of bacteria (**Figure 2B**). Attenuated *L. interrogans* serovar Pomona tend to bind less to purified properdin than *L. interrogans* serovar Pomona and non-pathogenic *L. biflexa* serovar Patoc strain Patoc I. However, this was not statistically significant.

### *Leptospira* spp. Binds to Purified Properdin Independently of C3b

**Figure 3:**
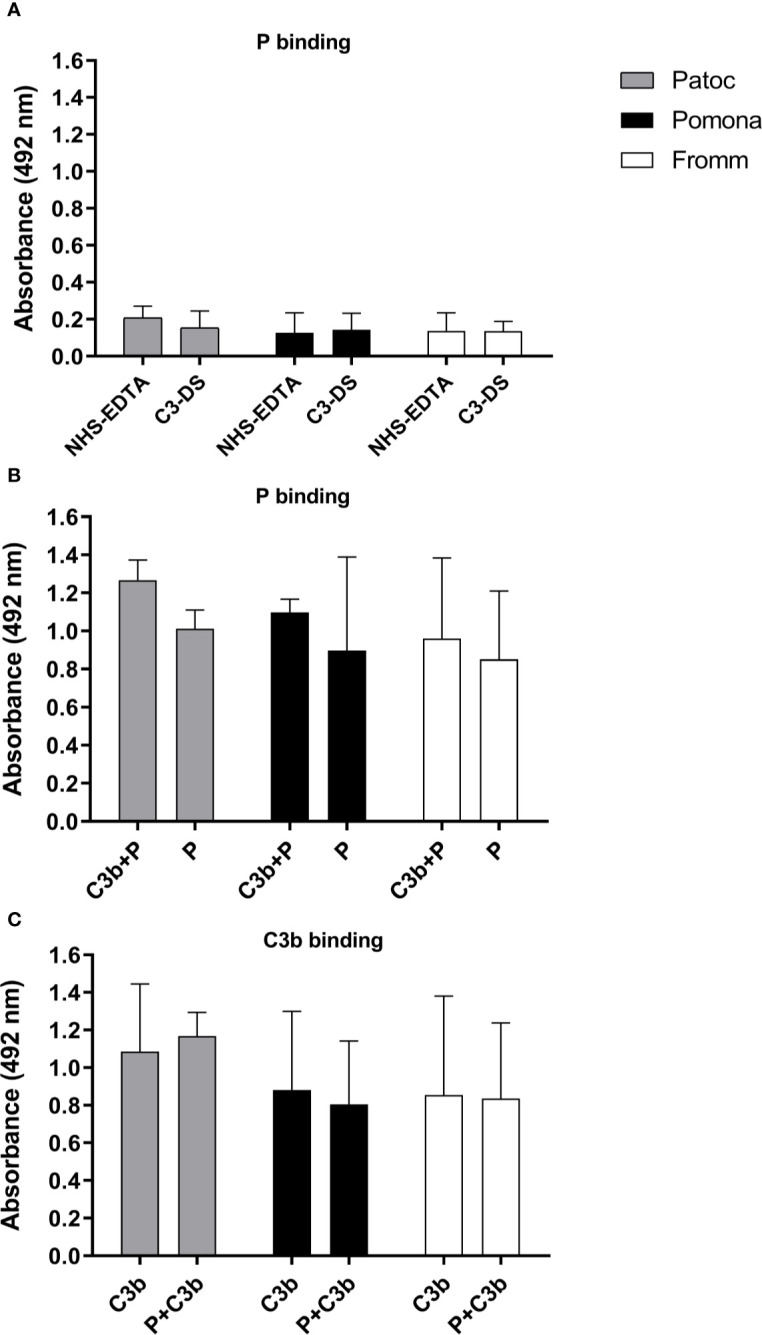
Binding of properdin to *Leptospira* occurs even in the absence of C3b. The binding of properdin (P) on *L. biflexa* serovar Patoc strain Patoc I (non-pathogenic), *L. interrogans* serovar Pomona strain Pomona (pathogenic, attenuated), and *L. interrogans* serovar Kennewicki strain Fromm (pathogenic, virulent) was quantified by ELISA using polyclonal anti-human properdin **(A, B)** or anti-human C3 **(C)**. **(A)** *Leptospira* strains were incubated with 40% normal human serum-treated with EDTA (NHS-EDTA) or with C3 depleted serum (C3-DS). **(B)** *Leptospira* strains were incubated with purified properdin (P) for 1h at 37°C, washed, and then with C3b (C3b+P) or PBS. **(C)** *Leptospira* strains were incubated with purified C3b for 1h at 37°C, washed, and then incubated with P (P + C3b) or PBS. Baseline values obtained without any addition of P or C3b were subtracted respectively for each type of leptospire. Data are expressed as the mean ( ± SD) of three independent experiments each one performed in triplicate.

To investigate if properdin could bind directly to *Leptospira* strains even in the absence of C3b, we incubated *L. biflexa* serovar Patoc strain Patoc I, *L. interrogans* serovar Pomona strain Pomona, and *L. interrogans* serovar Kennewicki strain Fromm with NHS-EDTA or C3-DS. Bound-properdin was detected by ELISA using anti-human properdin. As indicated in **Figure 3A**, all three *Leptospira* strains bound to serum properdin in relatively low amounts. When purified properdin was used a more pronounced interaction was observed, and properdin binding was observed in the presence or absence of C3b (**Figures 3B, C**). It is worth to mention that purified C3b is able to bind to the *Leptospira* membrane (20, 21).

These data suggest that properdin can bind directly to yet unknown leptospiral ligands regardless of the presence of C3b, which allows us to suspect that this complement regulatory protein may interact with pathogen molecular patterns and directly trigger AP activation, in addition to its known ability to act as a C3 convertase stabilizing molecule.

### Binding of Different Properdin Oligomers to *Leptospira*

**Figure 4:**
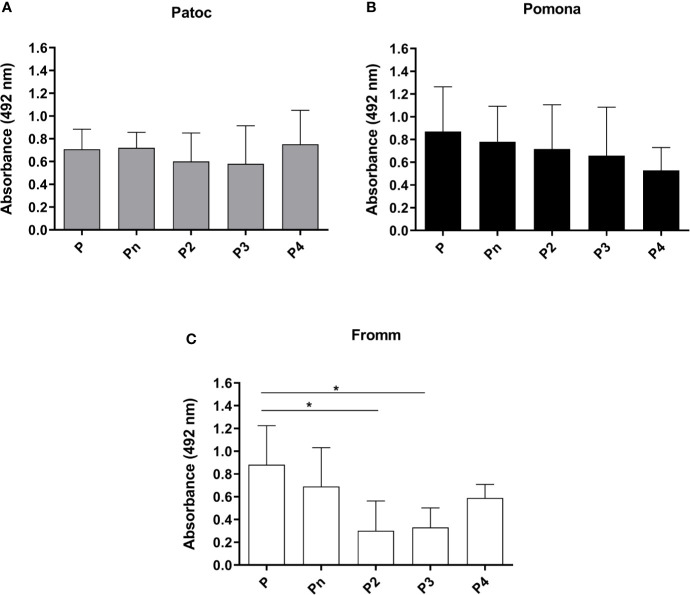
Binding of different properdin oligomers to pathogenic and non-pathogenic *Leptospira*. Unfractionated commercial properdin (P), high molecular weight aggregate not present in serum (Pn), properdin dimer (P2), trimer (P3), and tetramer (P4) were incubated with *L. biflexa* serovar Patoc strain Patoc I (non-pathogenic) **(A)**, *L. interrogans* serovar Pomona strain Pomona (pathogenic, attenuated) **(B)**), and *L. interrogans* serovar Kennewicki strain Fromm (pathogenic, virulent) **(C)**. Five µg of each properdin form were used and the binding on the *Leptospira* surface was evaluated by ELISA using polyclonal anti-human properdin. The mean and standard deviation correspond to three independent experiments, using two independent preparations of P2, P3, P4, or Pn. Baseline values obtained with PBS were subtracted, respectively for each type of leptospire. Data are expressed as the mean ( ± SD) of three independent experiments each one performed in triplicate. Statistically significant differences (ANOVA one-way test) are indicated. *p ≤ 0.05, confidence interval of 95%.

The interaction of *Leptospira* spp. with purified properdin oligomers (P2, P3, P4, and Pn) was investigated by comparing bacterial binding to each oligomer to that observed with commercial purified properdin. The results showed that all forms of properdin interact with the three strains of *Leptospira* tested. Virulent *L. interrogans* serovar Kennewicki strain Fromm binds less to P2 and P3 as compared to unfractionated commercial properdin (**Figure 4**). On the other hand, non**-**pathogenic *L. biflexa* serovar Patoc strain Patoc I and attenuated *L. interrogans* serovar Pomona strain Pomona bind similarly to unfractionated commercial properdin and to the properdin oligomers present in human serum.

### Interaction of Properdin With *Leptospira* OMPs

**Figure 5:**
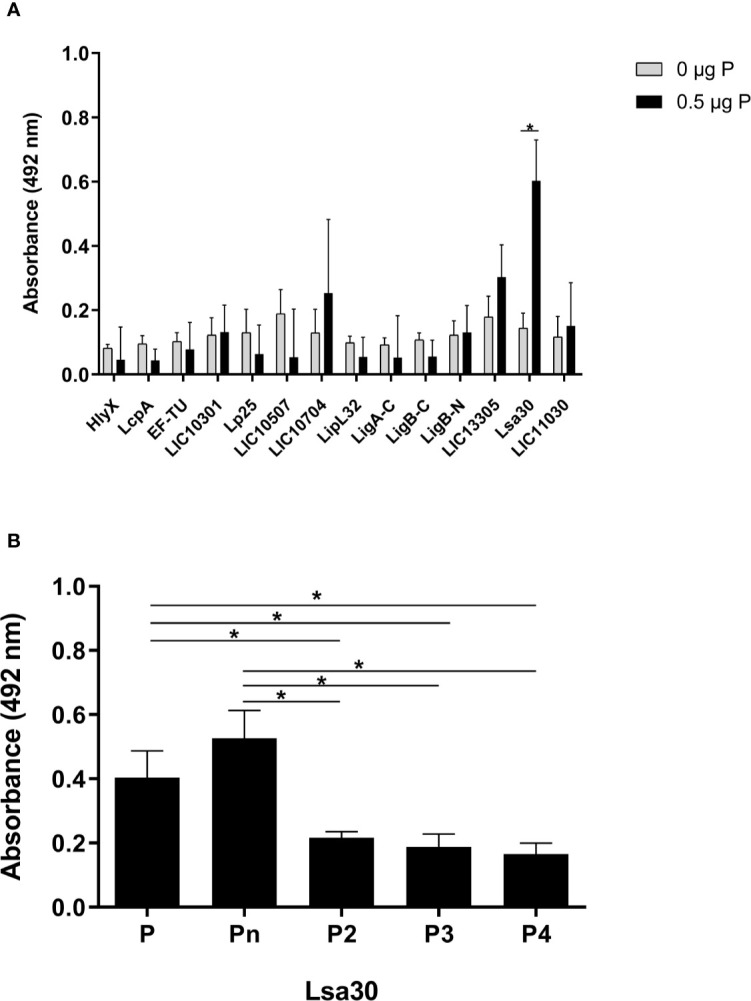
Interaction of outer membrane proteins of *Leptospira* with properdin. **(A)** Recombinant proteins of pathogenic *L. interrogans* serovar Copenhageni 10A (listed in **Table 1**) were immobilized on ELISA plate wells and incubated with 0.5 µg of unfractionated properdin (P). Binding was assessed by ELISA using polyclonal anti-human properdin. **(B)** Recombinant Lsa30 from pathogenic *L. interrogans* was immobilized and incubated with 0.5 µg of properdin (P), P2, P3, or P4. Binding was assessed as described above in **(A)**. These data represent the average ( ± SD) of three independent experiments, after subtracting the basal values obtained with PBS. Each experiment was performed in triplicate, using two independent preparations of P2, P3, P4, and Pn. Statistically significant differences (ANOVA one-way test) are indicated. *p ≤ 0.05, confidence interval of 95%.

A panel of recombinant proteins from pathogenic *L. interrogans* serovar Copenhageni 10A was used in an attempt to identify possible bacterial candidates for binding to properdin (**Table 1**). Regrettably, recombinant proteins from *L. biflexa* were not available. Among the proteins tested, Lsa30 (former LIC11087) was the only one which significantly bound to unfractionated commercial purified properdin (**Figure 5A**) as well as to P2, P3, P4, and Pn oligomers (**Figure 5B**). Binding of Lsa30 to Pn and to commercial purified properdin is more pronounced and significantly different from its binding to P2, P3 and P4.

### Formation of C3 Convertase on the *L. biflexa* Surface Requires the Presence of Properdin

**Figure 6:**
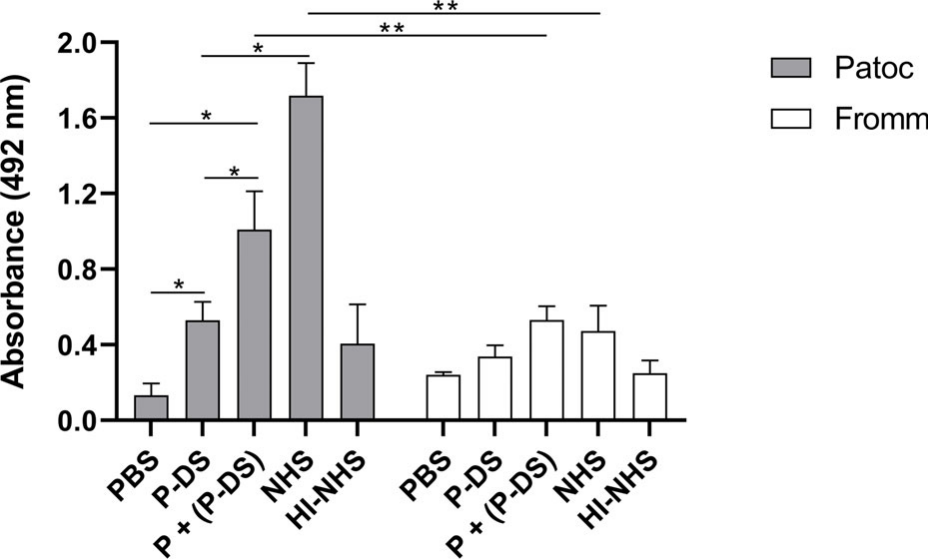
Properdin is required for AP activation on the surface of *L. biflexa* serovar Patoc strain Patoc I. Non-pathogenic *L. biflexa* serovar Patoc strain Patoc I or pathogenic *L. interrogans* serovar Kennewicki strain Fromm were incubated with properdin-depleted human serum (P-DS), normal human serum (NHS), or heat-inactivated NHS (HI-NHS). Where indicated, leptospires were previously treated with 5 µg of purified properdin (P) before incubating with P-DS [(P + (P-DS)]. The presence of the C3 convertase was evaluated by ELISA using polyclonal anti-Factor B Data are expressed as the mean ( ± SD) of three independent experiments each one performed in triplicate. Statistically significant differences (ANOVA one-way test) are indicated. *p ≤ 0.05; **p ≤ 0.001, confidence interval of 95%.

To assess if the formation of the C3 convertase (C3bBb) was stabilized by properdin (PC3bBb) on the leptospiral surface, *L. biflexa* serovar Patoc strain Patoc I and *L. interrogans* serovar Kennewicki strain Fromm were incubated under different serum conditions and the C3 convertase formation was detected by ELISA using anti-human Factor B antibody. As expected, C3 convertase formation on non-pathogenic *L. biflexa* serovar Patoc strain Patoc I treated with P-DS was limited and not significantly different when in the presence of HI-NHS (negative control) (**Figure 6**). However, when purified properdin was added to P-DS, a significant increase in the formation of C3bBb was observed, albeit at lower levels than that observed with NHS. With regard to the virulent *L. interrogans* serovar Kennewicki strain Fromm, no significant C3bBb formation was observed. This is probably because pathogenic leptospires bind quite well to Factor H and are able to evade complement activation (3, 5, 19).

## Discussion

The positive regulatory protein properdin stabilizes the C3 convertase and C5 convertase formed by the AP [(15, 16), and reviewed in (10)]. Its relevance is even more evident in immunodeficient patients that lack properdin since they are more susceptible to repeated infections caused by *Neisseria meningitidis* (22) and often results in pneumonia and otitis media (23, 24).

Since Pillemer’s early work in 1954 (13), it is known that properdin can recognize pathogenic targets and thus activates the complement system (14). We are unaware of studies in the literature that decipher the role of properdin during infection with leptospires. The relevance of properdin in the elimination of non-pathogenic leptospires was confirmed in this study. As previously described (3, 4), *L. biflexa* serovar Patoc strain Patoc I is rapidly lysed in the presence of NHS. Here we have shown that approximately 70% of the cells of strain Patoc I survived in P-DS. Recently, we have demonstrated that both the Alternative and Lectin Pathways contribute to the killing of *L. biflexa* serovar Patoc (19). This could explain why, even in the absence of PC3bBb, a residual killing activity is still observed in the presence of P-DS. As purified properdin was added (up to physiological levels) to this depleted serum, the complement-mediated bactericidal activity was restored in a dose-dependent manner, confirming the importance of this regulatory protein for the control of saprophytic *Leptospira* infection. On the other hand, pathogenic *Leptospira* are more resistant to complement activation since they exhibit several immune evasion mechanisms [reviewed in (25)].

Properdin binds with similar affinity to non-pathogenic leptospires and as well to virulent or attenuated pathogenic species. In addition, purified properdin binds more efficiently to these bacteria compared to human serum properdin possibly because of competition with other serum molecules that could target the same interaction sites on the *Leptospira* surface.

Interestingly, properdin can bind directly to the surface of *Leptospira*, even in the absence of C3b fragments, suggesting that properdin may interact directly with molecular patterns present on the surface of this spirochete. Our results reinforce the existence of two models (direct and indirect) of properdin to participate in the activation of the AP, since properdin can bind to *Leptospira* before and after generation of activated C3b fragments. This observation is in agreement with previous results published by various investigators that demonstrated that properdin can directly bind to *Neisseria gonorrhoeae*, *Chlamydia pneumoniae*, apoptotic and necrotic cells, rabbit erythrocytes, and zymosan particles (26–31). Thus, we suggest that properdin may participate in the recognition of certain microorganism patterns through surface receptors not yet identified. However, this hypothesis is controversial and not completely endorsed by the work of Agarwal and colleagues (32) who did not observe direct binding to *Neisseria* when using physiological forms of properdin. In addition, the binding of physiological forms of properdin to erythrocytes or zymosan was not observed (27). No significant binding of properdin to human endothelial cells (HUVECs), zymosan from *Saccharomyces cerevisae* and *Escherichia coli* was detected in the presence of compstatin Cp40 (which inhibits C3 cleavage) or C3-DS (33).

Previous studies emphasized the importance of using isolated forms of properdin present in serum, since after the purification process there is formation of high molecular weight properdin (Pn) aggregates that are absent under physiological conditions, and these aggregates could *per se* induce complement activation in the fluid phase (26, 27). In this study, we demonstrate that non-pathogenic and pathogenic leptospires can bind to all properdin oligomers, as well as to unfractionated properdin and Pn form. However, pathogenic leptospires interacted less strongly with P2, P3, and P4 oligomers than with unfractionated properdin and its Pn form. We emphasize that the binding of P2 and P3 to pathogenic leptospires was not significantly different when compared to the negative control. Since P2 and P3 are the most abundant forms of circulating properdin, reduced binding of these two forms to pathogenic *L. interrogans* would diminish AP activation on the surface of these bacteria, thus allowing them to evade complement-mediated host defense.

The kidneys are one of the main target organs for leptospiral colonization, and it has been shown that properdin binds to renal proximal tubule HK-2 cells (34). Thus, we speculate that properdin binding to pathogenic leptospires and to renal cells could be in some extent important for invasion and subsequent dissemination through contaminated urine. This hypothesis remains to be investigated.

As we have been focusing our research efforts on characterizing *Leptospira* virulence factors over the last years, we had a panel of *L. interrogans* serovar Copenhageni 10A recombinant proteins at our disposal. Their possible interaction with properdin was then evaluated. Only Lsa30, a ~32 kDa membrane protein, also known as LIC11087, bound to properdin.

Lsa30 interacted with properdin P2, P3, and P4 as well as to the Pn form. However, the Lsa30 interaction with properdin oligomers P2, P3, and P4 was significantly lower when compared to its interaction with properdin in unfractionated form and Pn. These results are consistent with those observed for pathogenic leptospires, which did not bind significantly to fractionated properdin forms.

Once we had confirmed a direct interaction of properdin with different leptospiral strains, we evaluated whether the properdin bound on the spirochete surface would be able to promote activation of the AP. Pathogenic leptospires showed little or no activation of the AP in the presence of properdin on its surface. This effect can be attributed to: *i)* pathogenic leptospires and Lsa30 do not bind consistently to serum properdin oligomers and, *ii)* pathogenic bacteria interact with Factor H, and negatively regulate complement activation on their surface (3–5).

In response to host defense mechanisms, pathogenic microorganisms express various virulence mechanisms. *Leptospira* evasion mechanisms that directly inhibit properdin function are not yet known. However, a new mechanism was described by Tsao et al. (35) who demonstrate that exotocin B (SPE B), a pyrogenic streptococcal cysteine protease, was able to degrade properdin, impairing the activation of the AP. As a consequence, opsophagocytosis by neutrophils was affected, preventing the death of bacteria by neutrophils. This protease also contributes to the degradation of fibronectin, vitronectin and fibrinogen, and helps the pathogen to evade host defenses and increase its replication. Furthermore, S20NS protein, a tick salivary protein, interacts with properdin and, upon binding, this positive regulator dissociates from C3 convertase, inhibiting complete activation of the AP (36). Evasion of complement by pathogenic leptospires by secretion of proteases that cleave important complement components (C3, C3b, iC3b, C2, and C4) was also observed by our group (37). We evaluated whether such proteases would be able to cleave properdin, and we observed that properdin degradation only occurred under conditions of low specificity with an unsuitable substrate enzyme ratio (data not shown).

Neutrophils are phagocytes rapidly attracted in large numbers to infection sites and are considered an important source of properdin release after inflammatory stimuli such as LPS, C5a and inflammatory cytokines (28, 38). We wondered if neutrophils could internalize more leptospires when coated with properdin, effectively contributing to their elimination at the site of infection. Some studies suggest that the evasion by pathogenic bacteria of the human macrophage cell line (THP-1) from the phagolysosome may be another mechanism presented by these spirochetes to evade innate immunity and later colonize target organs of the cell host (39).

The results presented here contribute to our understanding of the role of properdin in direct interactions with leptospires. Properdin interacts directly or indirectly with pathogenic, attenuated and non-pathogenic leptospires. However, the most abundant forms of properdin present in serum (P2 and P3) do not bind pathogenic leptospires significantly. Thus, we suggest that properdin, besides being a positive regulator of the C3 convertase of the AP, could also participate in the recognition of molecular patterns of *Leptospira* and promote the activation of the complement by the AP. Studies aimed at identifying whether properdin can bind directly to leptospires *in vivo* and play a significant role in infectivity are warranted.

## Data Availability Statement

The raw data supporting the conclusions of this article will be made available by the authors, without undue reservation.

## Author Contributions

AG and LI designed the experiments. AG performed the experiments. GS and VF provided the fractioned purified properdin forms. SA provided the bacterial cultures. PH, PA, and AB provided the *Leptospira* recombinant proteins. AG, AB, and LI wrote the manuscript. All authors contributed to the article and approved the submitted version.

## Funding

This work was supported by Fundação de Amparo à Pesquisa do Estado de São Paulo (FAPESP), Brazil (Proc # 2017/12924-3)). AG received student fellowships from FAPESP (2012/23708-6) and Conselho Nacional de Pesquisa e Desenvolvimento Científico (CNPq), Brazil (Proc. 141874/2012-0).

## Conflict of Interest

The authors declare that the research was conducted in the absence of any commercial or financial relationships that could be construed as a potential conflict of interest.

## References

[1] Vincent AT Schiettekatte O Goarant C Neela VK Bernet E Thibeaux R Revisiting the taxonomy and evolution of pathogenicity of the genus *Leptospira* through the prism of genomics. PLoS Negl Trop Dis (2019) 13(5):e0007270. 10.1371/journal.pntd 31120895 PMC6532842

[2] Fraga TR Isaac L Barbosa AS Complement Evasion by Pathogenic *Leptospira.* Front. Immunol (2016) 7:623:623. 10.3389/fimmu.2016.00623 PMC5174078 28066433

[3] Meri T Murgia R Stefanel P Meri S Cinco M Regulation of complement activation at the C3-level by serum resistant leptospires. Microb Pathog (2005) 39:139–47. 10.1016/j.micpath.2005.07.003 16169184

[4] Barbosa AS Abreu PA Vasconcellos AS Morais ZM Gonçales AP Silva AS Immune evasion of *Leptospira* spp. by acquisition of human complement regulator C4BP. Infect Immun (2009) 77:1137–43. 10.1128/IAI.01310-08 PMC2643629 19114549

[5] Castiblanco-Valencia MM Fraga TR Silva LB Monaris D Abreu PA Strobel S Leptospiral immunoglobulin-like proteins interact with human complement regulators factor H, FHL-1, FHR-1, and C4BP. J Infect Dis (2012) 205(6):995–1004. 10.1093/infdis/jir875 22291192

[6] Goundis D Reid KB Properdin, the terminal complement components, thrombospondin and the circumsporozoite protein of malaria parasites contain similar sequence motifs. Nature (1988) 335:82–5. 10.1038/335082a0 3045564

[7] Higgins JM Wiedemann H Timpl R Reid KB Characterization of mutant forms of recombinant human properdin lacking single thrombospondin type I repeats. Identification of modules important for function. J Immunol (1995) 155(12):5777–85. 7499866

[8] Nolan KF Kaluz S Higgins JM Goundis D Reid KB Characterization of the human properdin gene. Biochem J (1992) 287:291–7. 10.1042/bj2870291 PMC1133157 1417780

[9] Smith CA Pangburn MK Vogel CW Muller-Eberhard HJ Molecular architecture of human properdin, a positive regulator of the alternative pathway of complement. J Biol Chem (1984) 259(7):4582–8. 6707020

[10] Chen JY Cortes C Ferreira VP Properdin: A multifaceted molecule involved in inflammation and diseases. Mol Immunol (2018) 102:58–72. 10.1016/j.molimm.2018.05.018 29954621 PMC7375857

[11] de Paula PF Barbosa JE Junior PR Ferriani VP Latorre MR Nudelman V Ontogeny of complement regulatory proteins - concentrations of factor h, factor I, C4b-binding protein, properdin and vitronectin in healthy children of different ages and in adults. Scand J Immunol (2003) 58(5):572–7. 10.1046/j.1365-3083.2003.01326.x 14629629

[12] Kouser L Abdul-Aziz M Nayak A Stover CM Sim RB Kishore U Properdin and factor H: opposing players on the alternative complement pathway “see-saw”. Front Immunol (2013) 23:93(4):93. 10.3389/fimmu.2013.00093 PMC3632793 23630525

[13] Pillemer L Blum L Lepow IH Ross OA Todd EW Wardlaw AC The properdin system and immunity: I. Demonstration and isolation of a new serum protein, properdin, and its role in immune phenomena. Science (1954) 120:279–85. 10.1126/science.120.3112.279 13186838

[14] Fearon DT Regulation of the amplification C3 convertase of human complement by an inhibitory protein isolated from human erythrocyte membrane. Proc Natl Acad Sci USA (1979) 76(11):5867–71. 10.1073/pnas.76.11.5867

[15] Pangburn MK Müller-Eberhard HJ The alternative pathway of complement. Springer Semin Immunopathol (1984) 7(2-3):163–92. 10.1007/BF01893019 6238433

[16] Fearon DT Austen KF Properdin: binding to C3b and stabilization of the C3b dependent C3 convertase. J Exp Med (1975) 142:856–63. 10.1084/jem.142.4.856 PMC2189935 1185108

[17] Barbosa AS Abreu PA Neves FO Atzingen MV Watanabe MM Vieira ML A newly identified leptospiral adhesin mediates attachment to laminin. Infect Immun (2006) 74(11):6356–64. 10.1128/IAI.00460-06 PMC1695492 16954400

[18] Moreno-Torres A Malvido-Jiménez IR Peña-Moctezuma A Castillo Sánchez LO Fraga TR Barbosa AS Culture-attenuated pathogenic *Leptospira* lose the ability to survive to complement-mediated-killing due to lower expression of factor H binding proteins. Microbes Infect (2019) 21(8-9):377–85. 10.1016/j.micinf.2019.03.001 30923000

[19] Alves da Silva PYO Midon LM Heinemann MB de Moraes Vasconcelos D Barbosa AS Isaac L Contribution of Complement System pathways to the killing of *Leptospira* spp. Microbes Infect (2020) S1286-4579(20):30143–X. 10.1016/j.micinf.2020.07.005 32730816

[20] Choy HA Multiple activities of LigB potentiate virulence of *Leptospira* interrogans: inhibition of alternative and classical pathways of complement. PloS One (2012) 7(7):e41566. 10.1371/journal.pone.0041566 22911815 PMC3402383

[21] Castiblanco-Valencia MM Fraga TR Pagotto AH Serrano SM Abreu PA Barbosa AS Plasminogen cleaves fibrinogen and the human complement proteins C3b and C5 in the presence of *Leptospira interrogans* proteins: A new role of LigA and LigB in invasion and complement immune evasion. Immunobiology (2016) 221(5):679–89. 10.1016/j.imbio.2016.01.001 26822552

[22] Sjöholm AG Inherited complement deficiency states: implications for immunity and immunological disease. APMIS (1990) 98(10):861–74. 10.1111/j.1699-0463.1990.tb05008.x 2147105

[23] Schejbel L Rosenfeldt V Marquart H Valerius NH Garred P Properdin deficiency associated with recurrent otitis media and pneumonia, and identification of male carrier with Klinefelter syndrome. Clin Immunol (2009) 131:456–62. 10.1016/j.clim.2009.02.008 19328743

[24] Skattum L van Deuren M van der Poll T Truedsson L Complement deficiency states and associated infections. Mol Immunol (2011) 48(14):1643–55. 10.1016/j.molimm.2011.05.001 21624663

[25] Barbosa AS Isaac L Complement Immune Evasion by Spirochetes. Curr Top Microbiol Immunol (2018) 415:215–38. 10.1007/82_2017_47 28939965

[26] Cortes C Ferreira VP Pangburn MK Native properdin binds to *Chlamydia pneumoniae and* promotes complement activation. Infect Immun (2011) 79(2):724–31. 10.1128/IAI.00980-10 PMC3028849 21134964

[27] Ferreira VP Cortes C Pangburn MK Native polymeric forms of properdin selectively bind to targets and promote activation of the alternative pathway of complement. Immunobiology (2010) 215(11):932:40. 10.1016/j.imbio.2010.02.002 20382442 PMC2949450

[28] Kemper C Hourcade DE Properdin: New roles in pattern recognition and target clearance. Mol Immunol (2008) 45(16):4048–56. 10.1016/j.molimm.2008.06.034 PMC2628304 18692243

[29] Kemper C Mitchell LM Zhang L Hourcade DE The complement protein properdin binds apoptotic T cells and promotes complement activation and phagocytosis. Proc Natl Acad Sci USA (2008) 105(26):9023–8. 10.1073/pnas.0801015105 PMC2449358 18579773

[30] Spitzer D Mitchell LM Atkinson JP Hourcade DE Properdin can initiate complement activation by binding specific target surfaces and providing a platform for de novo convertase assembly. J Immunol (2007) 179:2600–8. 10.4049/jimmunol.179.4.2600 17675523

[31] Xu W Berger SP Trouw LA de Boer HC Schlagwein N Mutsaers C Properdin binds to late apoptotic and necrotic cells independently of C3b and regulates alternative pathway complement activation. J Immunol (2008) 180(11):7613–21. 10.4049/jimmunol.180.11.7613 18490764

[32] Agarwal S Ferreira VP Cortes C Pangburn MK Rice PA Ram S An evaluation of the role of properdin in alternative pathway activation on *Neisseria meningitidis* and *Neisseria gonorrhoeae* . J Immunol (2010) 185(1):507–16. 10.4049/jimmunol.0903598 PMC2933790 20530262

[33] Harboe M Johnson C Nymo S Ekholt K Schjalm C Lindstad JK Properdin binding to complement activating surfaces depends on initial C3b deposition. Proc Natl Acad Sci USA (2017) 114(4):E534–9. 10.1073/pnas.1612385114 PMC5278493 28069958

[34] Gaarkeuken H Siezenga MA Zuidwijk K van Kooten C Rabelink TJ Daha MR Complement activation by tubular cells is mediated by properdin binding. Am J Physiol Renal Physiol (2008) 295:F1397–403. 10.1152/ajprenal.90313.2008 18753294

[35] Tsao N Tsai WH Lin YS Chuang WJ Wang CH Kuo CF Streptococcal pyrogenic exotoxin B cleaves properdin and inhibits complement-mediated opsonophagocytosis. Biochem Biophys Res Commun (2006) 339(3):779–84. 10.1016/j.bbrc.2005.11.078 16329996

[36] Tyson KR Elkins C de Silva AM A novel mechanism of complement inhibition unmasked by a tick salivary protein that binds to properdin. J Immunol (2008) 180(6):3964–8. 10.4049/jimmunol.180.6.3964 18322205

[37] Fraga TR Courrol D dos S Castiblanco-Valencia MM Hirata IY Vasconcellos SA Immune evasion by pathogenic *Leptospira* strains: the secretion of proteases that directly cleave complement proteins. J Infect Dis (2014) 209(6):876–86. 10.1093/infdis/jit569 24163418

[38] Kemper C Atkinson JP Hourcade DE Properdin: emerging roles of a pattern-recognition molecule. Ann Rev Immunol (2020) 28:131–55. 10.1146/annurev-immunol-030409-101250 19947883

[39] Li S Ojcius DM Liao S Li L Xue F Dong H Replication or death: distinct fates of pathogenic Leptospira strain Lai within macrophages of human or mouse origin. Innate Immun (2010) 16(2):80–92. 10.1177/1753425909105580 19587003

